# Promoting the use of personally relevant stimuli for investigating patients with disorders of consciousness

**DOI:** 10.3389/fpsyg.2015.01102

**Published:** 2015-07-30

**Authors:** Fabien Perrin, Maïté Castro, Barbara Tillmann, Jacques Luauté

**Affiliations:** ^1^Auditory Cognition and Psychoacoustics Team, Lyon Neuroscience Research Center (UCBL, CNRS UMR5292, Inserm U1028)Lyon, France; ^2^Henry Gabrielle Hospital, Hospices Civils de LyonLyon, France; ^3^Neurological Hospital, Hospices Civils de LyonLyon, France; ^4^IMPACT, Lyon Neuroscience Research Center (UCBL, CNRS UMR5292, Inserm U1028)Lyon, France

**Keywords:** disorders of consciousness, music, autobiographical memory, self-processing, internal and external networks, vegetative state, minimally conscious state, coma

## Abstract

Sensory stimuli are used to evaluate and to restore cognitive functions and consciousness in patients with a disorder of consciousness (DOC) following a severe brain injury. Although sophisticated protocols can help assessing higher order cognitive functions and awareness, one major drawback is their lack of sensitivity. The aim of the present review is to show that stimulus selection is crucial for an accurate evaluation of the state of patients with disorders of consciousness as it determines the levels of processing that the patient can have with stimulation from his/her environment. The probability to observe a behavioral response or a cerebral response is increased when her/his personal history and/or her/his personal preferences are taken into account. We show that personally relevant stimuli (i.e., with emotional, autobiographical, or self-related characteristics) are associated with clearer signs of perception than are irrelevant stimuli in patients with DOC. Among personally relevant stimuli, music appears to be a promising clinical tool as it boosts perception and cognition in patients with DOC and could also serve as a prognostic tool. We suggest that the effect of music on cerebral processes in patients might reflect the music’s capacity to act both on the external and internal neural networks supporting consciousness.

## Using Stimulation to Evaluate and Restore Cognitive Functions and Awareness in Disorder of Consciousness (DOC) Patients

Survivors of severe brain injury generally transit through different states of decrease or loss of awareness (both of their environment and of their self), named coma, vegetative state (VS) –or unresponsive wakefulness syndrome (UWS)– and minimally conscious state (MCS). Evaluating cognition, probing consciousness, and predicting recovery are critical clinical issues. Currently, repeated behavioral clinical examination provides the main approach to establish diagnosis in clinical practice. However, these behaviors are often fluctuating and their detection can be unreliable. Indeed, some studies have observed that up to 40% of MCS patients are erroneously diagnosed as vegetative by non-expert teams, this being explained by arousal fluctuation, motor disabilities, poor expertise in behavioral assessment, or the use of insensitive behavioral assessment scales (e.g., Schnakers et al., 2006). One additional important issue concerns the difficulty of the correct recognition of affective behaviors that is part of the definition of MCS (Giacino et al., 2002). Indeed, the detection of subtle, but potentially meaningful affective behaviors remains difficult and hard to differentiate from purely automatic responses. Moreover, a part of subjectivity and interpretation of behavioral responses is often difficult to avoid because of facing dramatic situations and family expectations. As misdiagnosis has consequences on treatment and end-of-life decisions, developing additional methods in order to improve the detection of signs of consciousness is therefore crucial.

Over the last 20 years, neurophysiological studies have been developed, both at rest or using sensory stimulations, to better evaluate the preserved cerebral functions in patients with disorders of consciousness (DOC). General brain network organization has been explored using functional connectivity analyses at rest, i.e., when patients are not perceptively solicited. This allows evaluating spontaneous brain activity, including networks that probably serve basic functions related to consciousness (Boly et al., 2008). Indeed, this method helps understanding whether information processing can be locally amplified and distributed on a long-distance, whole-brain scale (e.g., Tononi et al., 1994), with the latter one being probably a prerequisite for conscious access (Dehaene and Naccache, 2001). Using resting state functional magnetic resonance imaging connectivity analyses, it has been shown that the default mode network connectivity was negatively correlated with the degree of clinical consciousness impairment, ranging from MCS patients over VS patients to coma patients (Vanhaudenhuyse et al., 2010; Stender et al., 2014). Using ^15^O-radiolabeled water PET measurements of regional cerebral blood flow performed at rest, Silva et al. (2010) have also identified an impaired functional connectivity between the ascending reticular activating system and the precuneus during persistent VS.

Most frequently, evaluations rest on sensory stimulations. In this case, specific cerebral activations are investigated, from sensory detection to higher order cognitive processes, sometimes also including stimulus awareness. Most importantly, these neurophysiological studies have shown that patients with little or no behavioral evidence of conscious awareness may retain critical cognitive capacities (for a review, see for example Laureys and Schiff, 2012). For example, the primary and secondary auditory cortices are often activated by auditory stimuli in DOC patients. It has also been shown that an acoustically deviant stimulus (such as an infrequent high pitch tone compared to frequent low pitch tones) can evoke an early event-related potential (ERP) referred to as MisMatch Negativity (MMN), thus demonstrating that an automatic echoic memory can still be preserved in these patients (e.g., Fischer et al., 2010). Interestingly, the presence of the MMN is a predictor of awakening and precludes comatose patients from moving to a permanent VS (Fischer et al., 2004). Furthermore, the preservation of linguistic functions has been also investigated. While ERP studies showed that the cerebral signatures to the detection of incongruous speech, i.e., the N400 potential, is still observed in some VS and MCS patients (Schoenle and Witzke, 2004; Kotchoubey et al., 2005; Rämä et al., 2010; Steppacher et al., 2013), fMRI studies suggested that the cortical structures associated to speech processing (notably the temporal lobe) are also activated in these patients (Schiff et al., 2005; Coleman et al., 2007; Fernández-Espejo et al., 2008).

More recently, active paradigms (i.e., paradigms in which patients are asked to follow the researcher’s instructions) have been developed to investigate the preservation of the patient’s voluntary brain functions. A new MMN paradigm has been developed to test the ability to actively maintain mental representations in working memory and to use these informations strategically, by measuring cerebral responses to violations of local or global temporal regularities (Bekinschtein et al., 2009). Faugeras et al. (2011) have shown that two patients who developed the neural signature of long-term violation detection showed unequivocal clinical signs of consciousness within the 3–4 days following the ERP recording. In another active paradigm, Schnakers et al. (2008) have evaluated the ability for DOC patients to count their own first name. They showed that the P300 potential, which indicates that the individual discriminates her/his own name (Perrin et al., 1999), was greater in an active condition than in a passive condition (i.e., without any instruction to count) in both controls and MCS patients. This finding suggests that MCS patients can direct active attention to an auditory target when asked to follow instructions. Finally, a neuroimaging study has also shown that a presumably VS patient was still able to understand and follow verbal instructions, such as “imagine you are walking around in your house” or “imagine you are playing tennis,” although clinical evaluation failed to detect her as conscious (Owen et al., 2006). However, the number of VS patients who could actually pass that test was extremely low: in a more recent study testing 54 DOC patients with this paradigm, only five patients were able to perform both mental activities on request and only one patient was able to associate these activities with a yes-or-no response and communicate with the researchers (Monti et al., 2010). Thus, although the achievement of this kind of sustained mental imagery tasks offers a specific criterion to detect patients that are conscious and highly functioning, one major drawback of this criterion is its lack of sensitivity. These tests cannot detect patients who are in the process of recovering awareness, but who are unable to understand complex verbal commands and/or to produce the mental effort required by sustained mental imagery tasks.

In addition to its use in evaluating the patient’s state and diagnosis, sensory stimulation has been also proposed as an intervention strategy to enhance the patient’s engagement with the immediate environment and to increase her/his consciousness level (Canedo et al., 2002). The background tenets for supporting stimulation initiatives include (a) the general fear that sensory deprivation connected with the virtual isolation of the person after severe brain injury may have additional negative effects on her/his condition (Oleson and Zubek, 1970) and (b) the view that the plasticity of the central nervous system could definitely benefit from a rich stimulation regimen (Canedo et al., 2002; Lombardi et al., 2002; Elliott and Walker, 2005). The use of sensory stimulation for DOC patients has gained popularity during the 1980s, but beliefs and opinions about its effectiveness vary substantially among health professionals. Canedo et al. (2002) and Bekinschtein et al. (2005) tested the effect of multisensory stimulation (auditory, visual, tactile, gustatory, and olfactory stimulation) on DOC patients. They both showed clear progress for the patients with consistent responses to stimulations and progressively the ability to respond to yes/no questions. Di Stefano et al. (2012) have also used multisensory stimulation and showed that familiar stimuli elicited a greater range of behavioral responses. However, other studies could not bring reliable evidence to support beneficial effects of multisensory stimulation (for reviews, see Formisano et al., 2001; Lombardi et al., 2002; Rigaux and Kiefer, 2003; Lancioni et al., 2010). Sensory stimulation approaches are thus still controversial, notably because of the absence of control conditions and/or statistical analyses, but also because the fatigability of these particular patients is not considered (Wood et al., 1992). In clinical practice, sensory regulation approaches are preferred over these sensory stimulation programs, that are programs in which salient and significant stimuli are delivered, but in which resting periods are also required.

## Personalized Stimuli Enhance the Probability to Observe a Response in DOC Patients

Most of the clinical tests are standardized protocols in which well-controlled stimuli (such as pure tones) are used. While neutral stimuli facilitate comparisons between patients’ data, they might also be associated with a high number of false negatives because they cannot personally engage patients with disorders of consciousness. In line with this hypothesis, it has been shown that, in contrast to neutral stimuli, personalized stimuli enhance the probability to observe a cerebral response in DOC patients.

Familiarity has been frequently used to capture the patient’s attention and to evoke an emotional reaction. For example, Bekinschtein et al. (2004) have shown, in one MCS patient, an extended brain activation of the emotional network (amygdala, insula, inferior frontal gyrus) in response to his mother’s voice reading a story, as compared to a non-familiar voice. Similarly, familiar faces (but not unfamiliar faces) succeed in eliciting activations in face-selective brain areas (with further limbic and cortical activations) in VS patients (Sharon et al., 2013). Personal significance also increases the probability to observe a brain response in DOC patients. Enhanced cognitive responses evoked by emotional and salient (personal) stimuli were first demonstrated with ERP methodology (for a review, see Perrin, 2004). While the P300 component to rare tones is observed only in a few patients (Harris and Hall, 1990; Yingling et al., 1990; Gott et al., 1991; Rappaport et al., 1991; De Giorgio et al., 1993; Glass et al., 1998), its probability of occurrence is enhanced with relevant and meaningful stimuli. Indeed, Signorino et al. (1997) have shown that the P300 to tones is more frequently observed when a short phrase spoken by a member of the family is presented simultaneously. Similarly, the P300 response is enhanced and can be analyzed, even on an individual level, when the deviant stimulus is not a tone stimulus but the patient’s own name. Perrin et al. (2006) have shown that, when the subject’s own name (SON) is presented in series of equiprobable unfamiliar first names, the P300 can be observed in all MCS patients, and in certain VS patients (see also the studies of Mazzini et al., 2001; Fischer et al., 2008; Höller et al., 2011; Risetti et al., 2013 in which the SON is used as a novel stimulus). As compared to the P300 to rare tones, the P300 to the SON is elicited more frequently (Cavinato et al., 2011). This is in agreement with behavioral assessments in which patients show more localization to the SON as compared to neutral sound (Cheng et al., 2013), and with fMRI studies in which a widespread activation in the temporal structures was observed for meaningful sounds (infant cries and SON) as compared to meaningless noise stimuli (Laureys et al., 2004).

The cerebral response following the presentation of the SON has been interpreted, in both healthy participants and DOC patients, as an index of a discriminative processing to a very salient and emotional word (Perrin et al., 1999, 2006) and might be associated to the enhancement of both top–down attentional and/or arousal mechanisms (for a review see Chennu and Bekinschtein, 2012), as well as of self-processing (Laureys et al., 2007). These functional hypotheses are supported by neuroimaging studies in which it has been shown that SON processing is associated, in controls and DOC patients, with regional cerebral blood flow changes in the superior temporal gyrus and in the medial (both in frontal and parietal) cortical structures (Laureys et al., 2004; Perrin et al., 2005; Northoff et al., 2006; Staffen et al., 2006; Di et al., 2007; Qin et al., 2010; Crone et al., 2013; Huang et al., 2014; Nicholas et al., 2014).

## Music Evokes Attention, Emotion, Autobiographical, and Self-Processing in Healthy Subjects

One of the most emotional and salient stimulus of our environment is probably music. Neuroimaging studies have shown that music listening activates a vast bilateral network related to attention, semantic processing, memory, and the sensori-motor system (for a review, see for example Zatorre, 2013), but also to emotion. Indeed, the entire limbic/paralimbic system, including the amygdala, the hippocampus, the parahippocampal gyrus, the nucleus accumbens, the ventral tegmental area, the anterior cingulate, and the orbitofrontal cortex have been shown to be activated when listening to music (for a review, Koelsch, 2010). Music also evokes autobiographical memories (e.g., Janata et al., 2007). Indeed, neuroimaging studies suggest that the dorsal medial prefrontal cortex (MPFC) plays a central role when we experience episodic memories that are triggered by familiar songs from our personal past. MPFC (as part of an internal network) might act in concert with more lateral brain structures, such as the lateral prefrontal and posterior cortices (as part of an external network) both in terms of overall responsiveness to familiar and autobiographically salient songs and tonality tracking and musical structure processing (Janata, 2009a).

Autobiographical memory is recognized as being multifaceted, containing a body of general knowledge (shared with other individuals), as well as unique experiences specific to an individual, which have been accumulated since childhood, and which allow ourselves to construct a feeling of identity and continuity (Conway and Pleydell-Pearce, 2000; Piolino et al., 2009). Tulving’s (1993) conception of memory emphasizes the episodic aspects of the self, defending the role of a phenomenological self in the construction and maintenance of subjective continuity in time and personal identity. Constructing autobiographical memories involves search, monitoring, and self-referential processes that are associated with activations in different regions in the prefrontal cortex (Cabeza and St Jacques, 2007). This is accordance with studies investigating music perception and emotion; these studies reported that the amount of activation in the MPFC depends on the likelihood of experiencing a chill while listening to self-selected music (Blood and Zatorre, 2001; Janata, 2009a).

Music perception thus requires complex processing, including numerous cognitive functions, some of them being specific to music, and others being shared with other materials or functions (Zatorre, 2005). Consequently, it has been proposed that music listening can convey beneficial effects on cognitive processes (e.g., Schellenberg, 2006; Thaut, 2010). For example, it has been shown that listening to music daily over a 2-month period leads to an enhancement of cognitive recovery (memory, attention, language) and mood, as well as long-term plastic changes in early sensory processing (as indexed by the MMN), after cerebral artery stroke (Särkämö et al., 2008). An enhancement of visual attention has been also described in patients with visual neglect when the tasks are performed while listening to preferred music relative to unpreferred music (Soto et al., 2009). Listening to a short musical excerpt improves subsequent linguistic syntax processing in patients with basal ganglia lesions (Kotz et al., 2005), patients with Parkinson disease (Kotz and Gunter, 2015) as well as in patients with developmental language disorders (Przybylski et al., 2013). Recently, it has been shown for conscious patients from intensive care units that music exposure resulted in greater reduction of sedation frequency, in comparison to usual care or noise-canceling conditions (Chlan et al., 2013). The beneficial effects of music have been attributed to the stimulation and recovery of cerebral networks required for the processing of different information (e.g., music and language), but also to the emotional characteristics of music, which are able to increase arousal (Thompson et al., 2001) and to activate the reward system (Salimpoor et al., 2011).

## Music Boosts Cognition in DOC Patients

From the research reviewed here above (but see also Bigand et al., 2015), music appears to be one of the best candidates to stimulate cerebral processes in DOC patients. This particular stimulus could engage patients and helps them to reach a higher level of cognition.

In DOC patients, music has been mostly used in therapy up to now. Formisano et al. (2001) have used active music therapy in 34 severe brain-injured patients. This music therapy approach consisted in activities related to musical improvisation between the patient and the therapist by singing or by playing different musical instruments, according to the vital functions, the neurological conditions and the motor abilities of the patients. Formisano et al. (2001) showed an improvement of the collaboration capacity of the patients and a reduction of undesired behaviors, such as inertia (reduced psychomotor initiative) or psychomotor agitation. Magee (2005, 2007) reported an increased participation of a VS patient following exposure to live music and to familiar songs, changing the diagnosis to MCS. Similar effects have been also described in other behavioral single-case studies (e.g., Bower et al., 2013). However, it is difficult to draw firm conclusions from these studies as they did not use quantified measures and were missing control conditions/groups (for a review, see Lancioni et al., 2010). Recently, the beneficial effect of music has been started to be investigated in more experimental approaches for DOC patients. For example, the effect of preferred music on performance to the Coma Recovery Scale-Revised (CRS-R) was compared to that of a control condition using a noise stimulus (a meaningless, non-aversive, acoustically matched sound stimulus) in six MCS patients (Verger et al., 2014). Qualitative and quantitative analyses showed in 66.6% of the assessments a better result for the music condition than for the control condition. Thus, this study suggests that preferred music, as compared to the control condition, has a beneficial effect on the cognitive abilities of MCS patients.

The effect of music therapy on physiological parameters has been also explored in DOC patients. Lee et al. (2011) have observed in a VS patient the enhancement of the cardiovascular activity following 14-day stimulation with a musical stimulus (i.e., Mahler’s Second Symphony). Autonomic changes with possible emotional value were also observed in both healthy controls and six VS patients while they passively listened to music samples (Riganello et al., 2010). Finally, alterations in oxygen saturation, breathing frequency and facial expression were found following preferred music in 30 DOC patients (Puggina et al., 2011).

Up to now, only few studies have used neurophysiological methodologies to assess the effect of music on DOC patients’ cerebral responses. Using power spectra analyses, O’Kelly et al. (2013) have shown an increased EEG amplitude in alpha and theta bands when listening to the patients’ preferred music in six VS and four MCS patients (see also Aldridge et al., 1990). To investigate auditory perception of comatose and post-comatose patients, Jones et al. (2000) measured the MMN responses following tones that were either played with complex synthesized instrumental timbres or with simple pure tones (Jones et al., 2000). It has been shown that the harmonically richer musical tones elicited an MMN more frequently and with larger amplitude than did the simple sine wave tones (Kotchoubey et al., 2003). Very recently, Castro et al. (2015) have demonstrated that music boosts cognition in comatose and post-comatose patients. In this study, the patient’s names were presented after either an excerpt of the patient’s preferred music (music condition) or a meaningless noise stimulus (control condition). Seven of the 13 patients showed a significant discriminative ERP (N200 and/or P300) to the patient’s own name, whereas, in the control condition, only one patient showed this response. Furthermore, the results showed that the presence/absence of the cerebral response was associated with, respectively, good/bad outcomes.

Recently, two neuroimaging studies have also explored the effect of music on the brain functioning of DOC patients. Okumura et al. (2014) showed that a famous, generally well-known music, which should thus probably be also familiar to the patient (‘Les Toreador’ from ‘Carmen’ Suite No. 1 by Bizet) activated the bilateral superior temporal gyri in 2/2 MCS and 1/5 VS patients. Interestingly, although DOC patients generally lose long-range functional connections, listening to music elicits increased functional connectivity, as compared to a noise condition, in regions belonging to the auditory network (see Heine et al., personal communication).

## The Effect of Music on Consciousness

The beneficial effects of music on cognitive functions of patients with DOC might be explained by an overall cortical arousal and/or an awareness enhancement. This is in agreement with “the arousal and mood hypothesis,” suggesting that the effect of music listening on cognitive abilities can be attributed to changes in listeners’ arousal and mood (Nantais and Schellenberg, 1999). The beneficial effects of music might be also associated to its “engagement” properties. Indeed, two types of brain networks interact when listening the music (Janata, 2009b). An external network and an internal network. The network for external engagement is generally associated with cognitive functions of language processing (including semantics), working memory, mental imagery, attention, etc. (for a review, see Cabeza and Nyberg, 2000). This network could be conceptualized as the perception/action cycle that implies the lateral posterior part of the brain in sensation and perception and the lateral anterior part in action (Fuster, 2009). The network for internal engagement is anticorrelated to the previous one (when one is activated, the other is generally deactivated) and encompasses mainly medial brain areas (Vanhaudenhuyse et al., 2011). It is engaged in various forms of self-referential (Wicker et al., 2003; Northoff et al., 2006) and autobiographical re-experiencing processes (Cabeza and St Jacques, 2007), which both support the “default-mode” hypothesis (Raichle et al., 2001). In summary, the internal network would be associated to awareness of the self, while the external network would be involved in awareness of environment (Vanhaudenhuyse et al., 2011). Both networks would be particularly coupled in listening to familiar or preferred music (for a review, see Janata, 2009b). This proposal of internal and external engagement when listening to music can be connected to the proposal of internal and external networks related to consciousness, providing a hypothesis for the neural basis of the beneficial effects of music in DOC patients. The connectivity within an external fronto-parietal network (i.e., between primary cortices and “higher-order” associative cortices), as well as the connectivity inside the nodes of an internal network (precuneus/posterior cingulate, mesas-frontal/anterior cingulate, and temporo-parietal cortices), has been shown to have a correlation that is decreasing with an increasing level of consciousness (Laureys and Schiff, 2012). Thus, it could be suggested that the effect of music on cerebral processes in DOC patients might reflect the music’s capacity to act on these two major networks supporting consciousness.

## Conclusion and Clinical Perspectives

This review strongly suggests that personally relevant stimuli are associated with enhanced behavioral responses and/or cerebral responses indicating perception in DOC patients, as compared to irrelevant or neutral stimuli. Preferred music appears to be a promising clinical tool, as it seems boosting some cognitive processes and/or awareness in patients with DOC, and could further serve as a prognostic tool as well. Future studies should identify what kind of cognitive and/or conscious processes could be enhanced following the presentation of preferred music, for example whether attention and awareness could be boosted in active paradigms or whether word processing be enhanced also for common names (as observed for the SON). Future studies should also disentangle a general effect of music (because of its acoustic and structural features) from its autobiographical effects (because of its emotional and meaningful contents in relation to the patients’ personal memory). The beneficial effects of the preferred music could be indeed due to its sound complexity (rhythm, melody, tempo, syntactic-like structural organization, etc.), as well as to familiarity, emotional, episodic/autobiographical, and self-related features. For example, it would be interesting to contrast familiar and unfamiliar music or to compare the effect of preferred music with either fast or slow tempo, as previous studies have shown that these features can modulate cognition (e.g., Gomez and Danuser, 2007; Nombela et al., 2013; Tillmann et al., 2014).

The challenge is also to incorporate personally relevant stimuli into standardized assessments that will be comparable across patients. This issue appears even more critical as one of the current diagnostic criteria for MCS is the detection of “affective behaviors that occur in contingent relation to relevant environmental stimuli and are not due to reflexive activity” (Giacino et al., 2002). Although this criterion is used to define MCS, the assessment of affective behavior is poorly addressed (in the supplementary items) by the CRS-R that is the current gold standard clinical scale. Moreover, the method to assess this very important criterion is based on the observation or the reports from family and clinician of affective behaviors like smiling, laughing, frowning, crying that occur spontaneously or in response to a specific stimulus. The incorporation of personally relevant stimuli in clinical practice is difficult, but it could be done with success: indeed, more and more hospitals now use SON protocols even though they request a personalized voice recording. For music, one perspective for future development would be to test whether similar effects could be obtained with familiar music (and not only with personally preferred music). If it is the case, then one could imagine developing (for a given country or culture) a set of musical pieces (selected per age group) that could be used to stimulate cognition in DOC patients before the behavioral or the neurophysiological assessments.

Finally, this review also point to a potential role of music in rehabilitation, i.e., for a long-term effect of music on cognition and consciousness in patients with DOC. It is not possible to assert that the presentation of the music was responsible for a long-term increase of the level of arousal and awareness, but it has been previously shown that music can induce long-term cognitive improvement. For example, for patients after cerebral stroke, listening to music daily during a 2-month period has improved significantly verbal memory and focused attention 6 months after (Särkämö et al., 2008, 2010). Furthermore, it has been previously demonstrated that listening music after neural damage can induce long-term plastic changes in early sensory processing, which, in turn, may facilitate the recovery of higher cognitive functions (Särkämö et al., 2010). Thus, these data encourage testing in future research the potential long-term role of music listening on cerebral plasticity in DOC patients.

## Conflict of Interest Statement

The authors declare that the research was conducted in the absence of any commercial or financial relationships that could be construed as a potential conflict of interest.

## References

[B1] Aldridge D. Gustorff D. Hannich H. J. (1990). Where am I? Music therapy applied to coma patients. *J. R. Soc. Med.* 83 345–346. 2380961 10.1177/014107689008300602 PMC1292679

[B2] Bekinschtein T. A. Dehaene S. Rohaut B. Tadel F. Cohen L. Naccache L. (2009). Neural signature of the conscious processing of auditory regularities. *Proc. Natl. Acad. Sci. U.S.A.* 106 1672–1677. 10.1073/pnas.0809667106 19164526 PMC2635770

[B3] Bekinschtein T. Leiguarda R. Armony J. Owen A. Carpintiero S. Niklison J. (2004). Emotion processing in the minimally conscious state. *J. Neurol. Neurosurg. Psychiatry* 75 788 10.1136/jnnp.2003.034876 PMC1763566 15090585

[B4] Bekinschtein T. Tiberti C. Niklison J. Tamashiro M. Ron M. Carpintiero S. (2005). Assessing level of consciousness and cognitive changes from vegetative state to full recovery. *Neuropsychol. Rehabil.* 15 307–322. 10.1080/09602010443000443 16350974

[B5] Bigand E. Tillmann B. Peretz I. Zatorre R. J. Lopez L. Majno M. (2015). “THE NEUROSCIENCES AND MUSIC V - Cognitive Stimulation and Rehabilitation,” in *Proceedings of the International Meeting held on May 29-June 1, 2014, in Dijon (*FRANCE*)*, Vol. 1337 New York: Annals of the New York Academy of Sciences, 271. 10.1111/nyas.12732 25773644

[B6] Blood A. J. Zatorre R. J. (2001). Intensely pleasurable responses to music correlate with activity in brain regions implicated in reward and emotion. *Proc. Natl. Acad. Sci. U.S.A.* 98 11818–11823. 10.1073/pnas.191355898 11573015 PMC58814

[B7] Boly M. Phillips C. Tshibanda L. Vanhaudenhuyse A. Schabus M. Dang-Vu T. T. (2008). Intrinsic brain activity in altered states of consciousness: how conscious is the default mode of brain function? *Ann. N. Y. Acad. Sci.* 1129 119–129. 10.1196/annals.1417.015 18591474 PMC2855379

[B8] Bower J. Catroppa C. Grocke D. Shoemark H. (2013). Music therapy for early cognitive rehabilitation post-childhood TBI: an intrinsic mixed methods case study. *Dev. Neurorehabil.* 17 339–346. 10.3109/17518423.2013.778910 23815784

[B9] Cabeza R. Nyberg L. (2000). Imaging cognition II: an empirical review of 275 PET and fMRI studies. *J. Cogn. Neurosci.* 12 1–47. 10.1162/08989290051137585 10769304

[B10] Cabeza R. St Jacques P. (2007). Functional neuroimaging of autobiographical memory. *Trends Cogn. Sci.* 11 219–227. 10.1016/j.tics.2007.02.005 17382578

[B11] Canedo A. Grix M. C. Nicoletti J. (2002). An analysis of assessment instruments for the minimally responsive patient (MRP): clinical observations. *Brain Inj.* 16 453–461. 10.1080/02699050110119853 12097227

[B12] Castro M. Tillmann B. Luauté J. Corneyllie A. Dailler F. André-Obadia N. (2015). Boosting cognition with music in patients with disorders of consciousness. *Neurorehabil. Neural Repair* 10.1177/1545968314565464 [Epub ahead of print] 25650390

[B13] Cavinato M. Volpato C. Silvoni S. Sacchetto M. Merico A. Piccione F. (2011). Event-related brain potential modulation in patients with severe brain damage. *Clin. Neurophysiol.* 122 719–724. 10.1016/j.clinph.2010.08.024 21051281

[B14] Cheng L. Gosseries O. Ying L. Hu X. Yu D. Gao H. (2013). Assessment of localisation to auditory stimulation in post-comatose states: use the patient’s own name. *BMC Neurol.* 13:27 10.1186/1471-2377-13-27 PMC3606124 23506054

[B15] Chennu S. Bekinschtein T. A. (2012). Arousal modulates auditory attention and awareness: insights from sleep, sedation, and disorders of consciousness. *Front. Psychol.* 3:65 10.3389/fpsyg.2012.00065 PMC3293189 22403565

[B16] Chlan L. L. Weinert C. R. Heiderscheit A. Tracy M. F. Skaar D. J. Guttormson J. L. (2013). Effects of patient-directed music intervention on anxiety and sedative exposure in critically ill patients receiving mechanical ventilatory support: a randomized clinical trial. *JAMA* 309 2335–2344. 10.1001/jama.2013.5670 23689789 PMC3683448

[B17] Coleman M. R. Rodd J. M. Davis M. H. Johnsrude I. S. Menon D. K. Pickard J. D. (2007). Do vegetative patients retain aspects of language comprehension? Evidence from fMRI. *Brain* 130(Pt 10), 2494–2507. 10.1093/brain/awm170 17827174

[B18] Conway M. A. Pleydell-Pearce C. W. (2000). The construction of autobiographical memories in the self-memory system. *Psychol. Rev.* 107 261–288. 10.1037/0033-295X.107.2.261 10789197

[B19] Crone J. S. Höller Y. Bergmann J. Golaszewski S. Trinka E. Kronbichler M. (2013). Self-related processing and deactivation of cortical midline regions in disorders of consciousness. *Front. Hum. Neurosci.* 7:504 10.3389/fnhum.2013.00504 PMC3752588 23986685

[B20] De Giorgio C. M. Rabinowicz A. L. Gott P. S. (1993). Predictive value of P300 event-related potentials compared with EEG and somatosensory evoked potentials in non-traumatic coma. *Acta Neurol. Scand.* 87 423–427. 10.1111/j.1600-0404.1993.tb04128.x 8333248

[B21] Dehaene S. Naccache L. (2001). Towards a cognitive neuroscience of consciousness: basic evidence and a workspace framework. *Cognition* 79 1–37. 10.1016/S0010-0277(00)00123-2 11164022

[B22] Di H. B. Yu S. M. Weng X. C. Laureys S. Yu D. Li J. Q. (2007). Cerebral response to patient’s own name in the vegetative and minimally conscious states. *Neurology* 68 895–899. 10.1212/01.wnl.0000258544.79024.d0 17372124

[B23] Di Stefano C. Cortesi A. Masotti S. Simoncini L. Piperno R. (2012). Increased behavioural responsiveness with complex stimulation in VS and MCS: preliminary results. *Brain Inj.* 26 1250–1256. 10.3109/02699052.2012.667588 22616735

[B24] Elliott L. Walker L. (2005). Rehabilitation interventions for vegetative and minimally conscious patients. *Neuropsychol. Rehabil.* 15 480–493. 10.1080/09602010443000506 16350989

[B25] Faugeras F. Rohaut B. Weiss N. Bekinschtein T. A. Galanaud D. Puybasset L. (2011). Probing consciousness with event-related potentials in vegetative state. *Neurology* 77 264–268. 10.1212/WNL.0b013e3182217ee8 21593438 PMC3136052

[B26] Fernández-Espejo D. Junqué C. Vendrell P. Bernabeu M. Roig T. Bargalló N. (2008). Cerebral response to speech in vegetative and minimally conscious states after traumatic brain injury. *Brain Inj.* 22 882–890. 10.1080/02699050802403573 18850346

[B27] Fischer C. Dailler F. Morlet D. (2008). Novelty P3 elicited by the subject’s own name in comatose patients. *Clin. Neurophysiol.* 119 2224–2230. 10.1016/j.clinph.2008.03.035 18760663

[B28] Fischer C. Luauté J. Adeleine P. Morlet D. (2004). Predictive value of sensory and cognitive evoked potentials for awakening from coma. *Neurology* 63 669–673. 10.1212/01.WNL.0000134670.10384.E2 15326240

[B29] Fischer C. Luaute J. Morlet D. (2010). Event-related potentials (MMN and novelty P3) in permanent vegetative or minimally conscious states. *Clin. Neurophysiol.* 121 1032–1042. 10.1016/j.clinph.2010.02.005 20202899

[B30] Formisano R. Vinicola V. Penta F. Matteis M. Brunelli S. Weckel J. W. (2001). Active music therapy in the rehabilitation of severe brain injured patients during coma recovery. *Ann. Ist. Super. Sanita* 37 627–630. 12046234

[B31] Fuster J. M. (2009). Cortex and memory: emergence of a new paradigm. *J. Cogn. Neurosci.* 21 2047–2072. 10.1162/jocn.2009.21280 19485699

[B32] Giacino J. T. Ashwal S. Childs N. Cranford R. Jennett B. Katz D. I. (2002). The minimally conscious state: definition and diagnostic criteria. *Neurology* 58 349–353. 10.1212/WNL.58.3.349 11839831

[B33] Glass I. Sazbon L. Groswasser Z. (1998). Mapping “cognitive” event-related potentials in prolonged postcoma unawareness state. *Clin. Electroencephalogr.* 29 19–30. 10.1177/155005949802900109 9472422

[B34] Gomez P. Danuser B. (2007). Relationships between musical structure and psychophysiological measures of emotion. *Emotion* 7 377–387. 10.1037/1528-3542.7.2.377 17516815

[B35] Gott P. S. Rabinowicz A. L. DeGiorgio C. M. (1991). P300 auditory event-related potentials in nontraumatic coma. Association with Glasgow Coma Score and awakening. *Arch. Neurol.* 48 1267–1270. 10.1001/archneur.1991.00530240071024 1845032

[B36] Harris D. P. Hall J. W. III. (1990). Feasibility of auditory event-related potential measurement in brain injury rehabilitation. *Ear Hear.* 11 340–350. 10.1097/00003446-199010000-00004 2262083

[B37] Höller Y. Kronbichler M. Bergmann J. Crone J. S. Schmid E. V. Golaszewski S. (2011). Inter-individual variability of oscillatory responses to subject’s own name. A single-subject analysis. *Int. J. Psychophysiol.* 80 227–235. 10.1016/j.ijpsycho.2011.03.012 21447360

[B38] Huang Z. Dai R. Wu X. Yang Z. Liu D. Hu J. (2014). The self and its resting state in consciousness: an investigation of the vegetative state. *Hum. Brain Mapp.* 35 1997–2008. 10.1002/hbm.22308 23818102 PMC6868996

[B39] Janata P. (2009a). The neural architecture of music-evoked autobiographical memories. *Cereb. Cortex* 19 2579–2594. 10.1093/cercor/bhp008 19240137 PMC2758676

[B40] Janata P. (2009b). “Music and the self,” in *Music That Works*, eds Haas R. Brandes V. (Wien: Springer), 131–141. 10.1007/978-3-211-75121-3_8

[B41] Janata P. Tomic S. T. Rakowski S. K. (2007). Characterization of music-evoked autobiographical memories. *Memory* 15 845–860. 10.1080/09658210701734593 17965981

[B42] Jones S. J. Vaz Pato M. Sprague L. Stokes M. Munday R. Haque N. (2000). Auditory evoked potentials to spectro-temporal modulation of complex tones in normal subjects and patients with severe brain injury. *Brain* 123(Pt 5), 1007–1016. 10.1093/brain/123.5.1007 10775545

[B43] Koelsch S. (2010). Towards a neural basis of music-evoked emotions. *Trends Cogn. Sci.* 14 131–137. 10.1016/j.tics.2010.01.002 20153242

[B44] Kotchoubey B. Lang S. Herb E. Maurer P. Schmalohr D. Bostanov V. (2003). Stimulus complexity enhances auditory discrimination in patients with extremely severe brain injuries. *Neurosci. Lett.* 352 129–132. 10.1016/j.neulet.2003.08.045 14625040

[B45] Kotchoubey B. Lang S. Mezger G. Schmalohr D. Schneck M. Semmler A. (2005). Information processing in severe disorders of consciousness: vegetative state and minimally conscious state. *Clin. Neurophysiol.* 116 2441–2453. 10.1016/j.clinph.2005.03.028 16002333

[B46] Kotz S. A. Gunter T. C. (2015). Can rhythmic auditory cuing remediate language-related deficits in Parkinson’s disease? *Ann. N. Y. Acad. Sci.* 1337 62–68. 10.1111/nyas.12657 25773618

[B47] Kotz S. A. Gunter T. C. Wonneberger S. (2005). The basal ganglia are receptive to rhythmic compensation during auditory syntactic processing: ERP patient data. *Brain Lang.* 95 70–71. 10.1016/j.bandl.2005.07.039

[B48] Lancioni G. E. Bosco A. Belardinelli M. O. Singh N. N. O’Reilly M. F. Sigafoos J. (2010). An overview of intervention options for promoting adaptive behavior of persons with acquired brain injury and minimally conscious state. *Res. Dev. Disabil.* 3 1121–1134. 10.1016/j.ridd.2010.06.019 20663643

[B49] Laureys S. Perrin F. Brédart S. (2007). Self-consciousness in non-communicative patients. *Conscious. Cogn.* 16 722–741. 10.1016/j.concog.2007.04.004 17544299

[B50] Laureys S. Perrin F. Faymonville M. E. Schnakers C. Boly M. Bartsch V. (2004). Cerebral processing in the minimally conscious state. *Neurology* 63 916–918. 10.1212/01.WNL.0000137421.30792.9B 15365150

[B51] Laureys S. Schiff N. D. (2012). Coma and consciousness: paradigms (re)framed by neuroimaging. *Neuroimage* 61 478–491. 10.1016/j.neuroimage.2011.12.041 22227888

[B52] Lee Y. C. Lei C. Y. Shih Y. S. Zhang W. C. Wang H. M. Tseng C. L. (2011). “HRV response of vegetative state patient with music therapy,” in *Proceedings of the Annual International Conference of the IEEE Engineering in Medicine and Biology Society, EMBC, 2011*, Boston, MA: IEEE. 10.1109/IEMBS.2011.6090488 22254653

[B53] Lombardi F. Taricco M. De Tanti A. Telaro E. Liberati A. (2002). Sensory stimulation for brain injured individuals in coma or vegetative state. *Cochrane Database Syst. Rev.* 2:CD001427 10.1002/14651858.cd001427 PMC7045727 12076410

[B54] Magee W. L. (2005). Music therapy with patients in low awareness states: approaches to assessment and treatment in multidisciplinary care. *Neuropsychol. Rehabil.* 15 522–536. 10.1080/09602010443000461 16350993

[B55] Magee W. L. (2007). Development of a music therapy assessment tool for patients in low awareness states. *NeuroRehabilitation* 22 319–324. 17971623

[B56] Mazzini L. Zaccala M. Gareri F. Giordano A. Angelino E. (2001). Long-latency auditory-evoked potentials in severe traumatic brain injury. *Arch. Phys. Med. Rehabil.* 82 57–65. 10.1053/apmr.2001.18076 11239287

[B57] Monti M. M. Vanhaudenhuyse A. Coleman M. R. Boly M. Pickard J. D. Tshibanda L. (2010). Willful modulation of brain activity in disorders of consciousness. *N. Engl. J. Med.* 362 579–589. 10.1056/NEJMoa0905370 20130250

[B58] Nantais K. M. Schellenberg E. G. (1999). The Mozart effect: an artifact of preference. *Psychol. Sci.* 10 370–373. 10.1111/1467-9280.00170

[B59] Nicholas C. R. McLaren D. G. Gawrysiak M. J. Rogers B. P. Dougherty J. H. Nash M. R. (2014). Functional neuroimaging of personally-relevant stimuli in a paediatric case of impaired awareness. *Brain Inj.* 28 1135–1138. 10.3109/02699052.2014.890745 24655307

[B60] Nombela C. Hughes L. E. Owen A. M. Grahn J. A. (2013). Into the groove: can rhythm influence Parkinson’s disease? *Neurosci. Biobehav. Rev.* 37 2564–2570. 10.1016/j.neubiorev.2013.08.003 24012774

[B61] Northoff G. Heinzel A. de Greck M. Bermpohl F. Dobrowolny H. Panksepp J. (2006). Self-referential processing in our brain: a meta-analysis of imaging studies on the self. *Neuroimage* 31 440–457. 10.1016/j.neuroimage.2005.12.002 16466680

[B62] O’Kelly J. James L. Palaniappan R. Taborin J. Fachner J. Magee W. L. (2013). Neurophysiological and behavioral responses to music therapy in vegetative and minimally conscious States. *Front. Hum. Neurosci.* 7:884 10.3389/fnhum.2013.00884 PMC3872324 24399950

[B63] Okumura Y. Asano Y. Takenaka S. Fukuyama S. Yonezawa S. Kasuya Y. (2014). Brain activation by music in patients in a vegetative or minimally conscious state following diffuse brain injury. *Brain Inj.* 28 944–950. 10.3109/02699052.2014.888477 24655034

[B64] Oleson D. S. Zubek J. P. (1970). Effect of one day of sensory deprivation on a battery of open-ended cognitive tests. *Percept. Mot. Skills* 31 919–923. 10.2466/pms.1970.31.3.919 5532110

[B65] Owen A. M. Coleman M. R. Boly M. Davis M. H. Laureys S. Pickard J. D. (2006). Detecting awareness in the vegetative state. *Science* 313 1402 10.1126/science.1130197 16959998

[B66] Perrin F. (2004). Auditory evoked potentials studies of information processing during human sleep. *Psychol. Belg.* 44 43–57.

[B67] Perrin F. García-Larrea L. Mauguière F. Bastuji H. (1999). A differential brain response to the subject’s own name persists during sleep. *Clin. Neurophysiol.* 110 2153–2164. 10.1016/S1388-2457(99)00177-7 10616121

[B68] Perrin F. Maquet P. Peigneux P. Ruby P. Degueldre C. Balteau E. (2005). Neural mechanisms involved in the detection of our first name: a combined ERPs and PET study. *Neuropsychologia* 43 12–19. 10.1016/j.neuropsychologia.2004.07.002 15488900

[B69] Perrin F. Schnakers C. Schabus M. Degueldre C. Goldman S. Brédart S. (2006). Brain response to one’s own name in vegetative state, minimally conscious state, and locked-in syndrome. *Arch. Neurol.* 63 562–569. 10.1001/archneur.63.4.562 16606770

[B70] Piolino P. Desgranges B. Eustache F. (2009). Episodic autobiographical memories over the course of time: cognitive, neuropsychological and neuroimaging findings. *Neuropsychologia* 47 2314–2329. 10.1016/j.neuropsychologia.2009.01.020 19524095

[B71] Przybylski L. Bedoin N. Krifi-Papoz S. Herbillon V. Roch D. Léculier L. (2013). Rhythmic auditory stimulation influences syntactic processing in children with developmental language disorders. *Neuropsychology* 27 121–131. 10.1037/a0031277 23356600

[B72] Puggina A. C. G. Paes da Silva M. J. Santos J. L. F. (2011). Use of music and voice stimulus on patients with disorders of consciousness. *J. Neurosci. Nurs.* 43 8–16. 10.1097/JNN.0b013e3182029778

[B73] Qin P. Di H. Liu Y. Yu S. Gong Q. Duncan N. (2010). Anterior cingulate activity and the self in disorders of consciousness. *Hum. Brain Mapp.* 31 1993–2002. 10.1002/hbm.20989 20336686 PMC6870855

[B74] Raichle M. E. MacLeod A. M. Snyder A. Z. Powers W. J. Gusnard D. A. Shulman G. L. (2001). A default mode of brain function. *Proc. Natl. Acad. Sci. U.S.A.* 98 676–582. 10.1073/pnas.98.2.676 11209064 PMC14647

[B75] Rämä P. Relander-Syrjänen K. Ohman J. Laakso A. Näätänen R. Kujala T. (2010). Semantic processing in comatose patients with intact temporal lobes as reflected by the N400 event-related potential. *Neurosci. Lett.* 474 88–92. 10.1016/j.neulet.2010.03.012 20226842

[B76] Rappaport M. McCandless K. L. Pond W. Krafft M. C. (1991). Passive P300 response in traumatic brain injury patients. *J. Neuropsychiatry Clin. Neurosci.* 3 180–185. 10.1176/jnp.3.2.180 1821233

[B77] Riganello F. Candelieri A. Quintieri M. Conforti D. Dolce G. (2010). Heart rate variability: an index of brain processing in vegetative state? An artificial intelligence, data mining study. *Clin. Neurophysiol.* 121 2024–2034. 10.1016/j.clinph.2010.05.010 20566300

[B78] Rigaux P. Kiefer C. (2003). Indications, effectiveness and tolerance of the rehabilitation techniques aimed at improving recovery of awareness following a traumatic brain injury. *Ann. Readapt. Med. Phys.* 46 219–226. 10.1016/S0168-6054(03)00082-5 12832137

[B79] Risetti M. Formisano R. Toppi J. Quitadamo L. R. Bianchi L. Astolfi L. (2013). On ERPs detection in disorders of consciousness rehabilitation. *Front. Hum. Neurosci.* 7:775 10.3389/fnhum.2013.00775 PMC3834290 24312041

[B80] Salimpoor V. N. Benovoy M. Larcher K. Dagher A. Zatorre R. J. (2011). Anatomically distinct dopamine release during anticipation and experience of peak emotion to music. *Nat. Neurosci.* 14 257–262. 10.1038/nn.2726 21217764

[B81] Särkämö T. Pihko E. Laitinen S. Forsblom A. Soinila S. Mikkonen M. (2010). Music and speech listening enhance the recovery of early processing after stroke. *J. Cogn. Neurosci.* 22 2716–2727. 10.1162/jocn.2009.21376 19925203

[B82] Särkämö T. Tervaniemi M. Laitinen S. Forsblom A. Soinila S. Mikkonen M. (2008). Music listening enhances cognitive recovery and mood after middle cerebral artery stroke. *Brain* 131 866–876. 10.1093/brain/awn013 18287122

[B83] Schellenberg E. G. (2006). “Exposure to music: the truth about the consequences,” in *The Child as Musician: A Handbook of Musical Development*, ed. McPherson G. (New York, NY: Oxford University Press), 111–134. 10.1093/acprof:oso/9780198530329.003.0006

[B84] Schiff N. D. Rodriguez-Moreno D. Kamal A. Kim K. H. Giacino J. T. Plum F. (2005). fMRI reveals large-scale network activation in minimally conscious patients. *Neurology* 64 514–523. 10.1212/01.WNL.0000150883.10285.44 15699384

[B85] Schnakers C. Giacino J. Kalmar K. Piret S. Lopez E. Boly M. (2006). Does the FOUR score correctly diagnose the vegetative and minimally conscious states? *Ann. Neurol.* 60 744–745. 10.1002/ana.20919 16847951

[B86] Schnakers C. Perrin F. Schabus M. Majerus S. Ledoux D. Damas P. (2008). Voluntary brain processing in disorders of consciousness. *Neurology* 71 1614–1620. 10.1212/01.wnl.0000334754.15330.69 19001251

[B87] Schoenle P. W. Witzke W. (2004). How vegetative is the vegetative state? Preserved semantic processing in VS patients–evidence from N 400 event-related potentials. *NeuroRehabilitation* 19 329–334. 15671587

[B88] Sharon H. Pasternak Y. Ben Simon E. Gruberger M. Giladi N. Krimchanski B. Z. (2013). Emotional processing of personally familiar faces in the vegetative state. *PLoS ONE* 8:e74711 10.1371/journal.pone.0074711 PMC3783455 24086365

[B89] Signorino M. D’Acunto S. Cercaci S. Pietropaoli P. Angeleri F. (1997). The P300 in traumatic coma: conditioning of the odd-ball paradigm. *J. Psychophysiol.* 11 59–70.

[B90] Silva S. Alacoque X. Fourcade O. Samii K. Marque P. Woods R. (2010). Wakefulness and loss of awareness: brain and brainstem interaction in the vegetative state. *Neurology* 74 313–320. 10.1212/WNL.0b013e3181cbcd96 20101037 PMC3462476

[B91] Soto D. Funes M. J. Guzmán-García A. Warbrick T. Rotshtein P. Humphreys G. W. (2009). Pleasant music overcomes the loss of awareness in patients with visual neglect. *Proc. Natl. Acad. Sci. U.S.A.* 106 6011–6016. 10.1073/pnas.0811681106 19307566 PMC2667081

[B92] Staffen W. Kronbichler M. Aichhorn M. Mair A. Ladurner G. (2006). Selective brain activity in response to one’s own name in the persistent vegetative state. *J. Neurol. Neurosurg. Psychiatry* 77 1383–1384. 10.1136/jnnp.2006.095166 17110753 PMC2077408

[B93] Stender J. Gosseries O. Bruno M. A. Charland-Verville V. Vanhaudenhuyse A. Demertzi A. (2014). Diagnostic precision of PET imaging and functional MRI in disorders of consciousness: a clinical validation study. *Lancet* 384 514–522. 10.1016/S0140-6736(14)60042-8 24746174

[B94] Steppacher I. Eickhoff S. Jordanov T. Kaps M. Witzke W. Kissler J. (2013). N400 predicts recovery from disorders of consciousness. *Ann. Neurol.* 73 594–602. 10.1002/ana.23835 23443907

[B95] Thaut M. H. (2010). Neurologic music therapy in cognitive rehabilitation. *Music Percept.* 27 281–285. 10.1525/mp.2010.27.4.281

[B96] Thompson W. F. Schellenberg E. G. Husain G. (2001). Arousal, mood, and the Mozart effect. *Psychol. Sci.* 12 248–251. 10.1111/1467-9280.00345 11437309

[B97] Tillmann B. Albouy P. Caclin A. Bigand E. (2014). Musical familiarity in congenital amusia: evidence from a gating paradigm. *Cortex* 59 84–94. 10.1016/j.cortex.2014.07.012 25151640

[B98] Tononi G. Sporns O. Edelman G. M. (1994). A measure for brain complexity: relating functional segregation and integration in the nervous system. *Proc. Natl. Acad. Sci. U.S.A.* 91 5033–5037. 10.1073/pnas.91.11.5033 8197179 PMC43925

[B99] Tulving E. (1993). “Self-knowledge of an amnesic individual is represented abstractly,” in *Mental Representation of Trait and Autobiographical Knowledge About the Self*, eds Srull T. K. Wyer R. S. (Hillsdale, NJ: Erlbaum), 147–157.

[B100] Vanhaudenhuyse A. Demertzi A. Schabus M. Noirhomme Q. Bredart S. Boly M. (2011). Two distinct neuronal networks mediate the awareness of environment and of self. *J. Cogn. Neurosci.* 23 570–578. 10.1162/jocn.2010.21488 20515407

[B101] Vanhaudenhuyse A. Noirhomme Q. Tshibanda L. J. Bruno M. A. Boveroux P. Schnakers C. (2010). Default network connectivity reflects the level of consciousness in non-communicative brain-damaged patients. *Brain* 133(Pt 1), 161–171. 10.1093/brain/awp313 20034928 PMC2801329

[B102] Verger J. Ruiz S. Tillmann B. Ben Romdhane M. De Quelen M. Castro M. (2014). Beneficial effect of preferred music on cognitive functions in minimally conscious state patients. *Rev. Neurol.* 170 693–699. 10.1016/j.neurol.2014.06.005 25287735

[B103] Wicker B. Ruby P. Royet J. P. Fonlupt P. (2003). A relation between rest and the self in the brain? *Brain Res. Brain Res. Rev.* 43 224–230. 10.1016/j.brainresrev.2003.08.003 14572916

[B104] Wood R. L. Winkowski T. B. Miller J. L. Tierney L. Goldman L. (1992). Evaluating sensory regulation as a method to improve awareness in patients with altered states of consciousness: a pilot study. *Brain Inj.* 6 411–418. 10.3109/02699059209008137 1393174

[B105] Yingling C. D. Hosobuchi Y. Harrington M. (1990). P300 as a predictor of recovery from coma. *Lancet* 336 873 10.1016/0140-6736(90)92372-O 1976890

[B106] Zatorre R. J. (2005). Neuroscience: finding the missing fundamental. *Nature* 436 1093–1094. 10.1038/4361093a 16121160

[B107] Zatorre R. J. (2013). Predispositions and plasticity in music and speech learning: neural correlates and implications. *Science* 342 585–589. 10.1126/science.1238414 24179219

